# Depression and Anxiety Among US Children and Young Adults

**DOI:** 10.1001/jamanetworkopen.2024.36906

**Published:** 2024-10-01

**Authors:** Anny H. Xiang, Mayra P. Martinez, Ting Chow, Sarah A. Carter, Sonya Negriff, Breda Velasquez, Joseph Spitzer, Juan Carlos Zuberbuhler, Ashley Zucker, Sid Kumar

**Affiliations:** 1 Department of Research & Evaluation, Kaiser Permanente Southern California, Pasadena; 2 Department of Child and Adolescent Psychiatry, Kaiser Permanente Southern California, Pasadena; 3 Division of Developmental Pediatrics, Kaiser Permanente Southern California, Pasadena; 4 Depression Care Management Program, Kaiser Permanente Southern California, Pasadena

## Abstract

**Importance:**

Data from surveys show increased mental health disorders in youths. However, little is known about clinical diagnosis over time.

**Objective:**

To assess the incidence, prevalence, and changes from 2017 to 2021 for depression and anxiety diagnosed clinically among children, adolescents, and young adults and to identify potential disparities.

**Design, Setting, and Participants:**

This cohort study included approximately 1.7 million individuals aged 5 to 22 years in Southern California. Data were extracted from electronic medical records; *International Statistical Classification of Diseases, Tenth Revision* (*ICD-10*), codes were used to identify depression and/or anxiety diagnosis for each study year from January 1, 2017, to December 31, 2021. Rates were stratified by age, gender, race and ethnicity, estimated household income, weight status, and comorbidity history. Changes over time and association with these variables were assessed using Poisson regression. Data were analyzed between June 1, 2022, and November 29, 2023.

**Main Outcomes and Measures:**

Clinical diagnosis of (1) depression and (2) anxiety without a depression diagnosis using *ICD-10* codes.

**Results:**

Among the 1.7 million participants, mean (SD) age was approximately 14 (5) years, and 51% were male. In terms of race and ethnicity for each study year, approximately 50% of participants were Hispanic; 8%, non-Hispanic Asian; 8%, non-Hispanic Black; and 23%, non-Hispanic White. From 2017 to 2021, depression diagnosis increased by 55.6% (from 1.35% to 2.10%) for incidence and 60.0% (from 2.55% to 4.08%) for prevalence; anxiety without depression diagnosis increased by 31.1% (from 1.77% to 2.32%) for incidence and 35.2% (from 3.13% to 4.22%) for prevalence (*P* < .001 for trend). The increases in rates were higher during the COVID-19 pandemic (2020-2021) than before the pandemic (2017-2019), except for depression incidence. Rates increased across all subgroups. Rates were highest for subgroups aged 14 to 17 and 18 to 22 years; female participants; those of non-Hispanic American Indian or Alaska Native, non-Hispanic White, or multiple races or ethnicities; and subgroups with higher household income, obesity (and underweight for anxiety without depression), or comorbidities. Among these factors, age was the most important factor for depression diagnosis, whereas weight status was the most important factor for anxiety without depression diagnosis.

**Conclusions and Relevance:**

This cohort study, using electronic medical record data from a large integrated health care system, found an increase in clinically diagnosed depression from 2017 to 2021, with a higher increase during the COVID-19 pandemic and higher rates in some subgroups. Equally important, this study identified high rates and an increase in clinical diagnosis of anxiety without a depression diagnosis. These results support the increased need in public health and health care effort to combat the mental health crisis in youths.

## Key Points

**Question:**

What are the incidence, prevalence, and changes from 2017 to 2021 for depression and anxiety diagnosed clinically among children, adolescents, and young adults and are there disparities among subgroups?

**Findings:**

In this cohort study of approximately 1.7 million individuals aged 5 to 22 years in Southern California, the overall incidence and prevalence of depression diagnosed clinically increased by approximately 60%, and anxiety diagnosed without depression incidence increased by 31% from 2017 to 2021. Rates increased across all subgroups and were greater during the COVID-19 pandemic.

**Meaning:**

These findings suggest the increased need for mental health services for youth.

## Teaser

This cohort study assesses changes in the incidence and prevalence of clinically diagnosed depression and anxiety without depression among US children, adolescents, and young adults aged 5 to 22 years from 2017 to 2021.

## Introduction

Depression and anxiety are serious mental health disorders affecting millions of US children.^1^ These disorders contribute an enormous burden on the health and well-being of individuals, families, and society, and have an estimated annual cost of $247 billion.^2^ The US Surgeon General has issued a health advisory about mental health in youths^3^ that includes a recommendation to better understand changes in incidence and prevalence.

Data on mental health conditions for US youths generally rely on 9 data systems that mostly consist of surveys, with 4 national surveys collecting parent- or self-reported depression and 1 national survey for anxiety.^1^ Data from the National Survey of Children’s Health (NSCH) study,^4^ a US nationally representative survey of randomly selected children, showed that depression among children and adolescents aged 3 to 17 years increased from 3.1% in 2016 to 4.0% in 2020, an approximately 30% increase over a 5-year period. The reported anxiety for this age group similarly increased from 7.1% in 2016 to 9.2% in 2020.^4^ A recent study^5^ using data from the 2021 National Survey on Drug Use and Health (NSDUH)^6^ showed that approximately 20% of US adolescents aged 12 to 17 years reported having major depressive disorder (MDD) in the past year; however, fewer than half reported receiving any mental health treatment, and rates varied greatly by race and ethnicity.

While data from these surveys provided important information about mental health conditions among US youths,^1,2,4,5,7^ little is known about the incidence and prevalence of and changes in depression and/or anxiety among US youths diagnosed from clinical care or the potential disparities covering both before and during the COVID-19 pandemic. It is important to assess this question, given the low treatment rate reported for MDD and racial and ethnic disparites.^5,6^ Clinically diagnosed depression includes depressive disorders that are characterized by sad or irritable mood or loss of interest in activities, causing significant impairment in daily life.^8^ Major depressive disorder is the most common condition for clinically diagnosed depression, with an episode of at least 2 weeks’ duration.^8,9,10^ Persistent depressive disorder or dysthymia is the next most common depressive disorder and is defined as a chronic depressed mood for at least 1 year in children and adolescents.^10,11^ Clinically diagnosed anxiety is a medical disorder with excessive feelings of worry or persistent, even intrusive thoughts about certain fears or constant fear in general that are developmentally inappropriate.^10,12^ Depression is a major comorbid condition of anxiety, with 20% to 70% lifetime risk of depression for patients with anxiety disorders.^13^ Thus, clinically diagnosed depression and/or anxiety captures severe conditions requiring medical interventions. Assessing the incidence and prevalence of and changes in clinically diagnosed depression and/or anxiety in children, adolescents, and young adults will help define the need for improving long-term health and well-being for our young population.

The purpose of this study was to assess trends of incidence and prevalence of clinically diagnosed depression and anxiety among individuals aged 5 to 22 years from 2017 to 2021. The age range covers the major educational time frame with the school social environment. Since many people with anxiety also have depression, we specifically reported anxiety without depression, a medical condition that has not been reported much by itself in prior studies. Anxiety involves feelings of excessive worry and nervousness, whereas depression involves feelings of hopelessness and reduced energy. These 2 conditions can occur at the same time; however, studies showed that anxiety disorders generally precede the presentation of MDD.^13,14^ Thus, reporting anxiety without depression in youths is important. Data were drawn from a population-based, large integrated health care system in Southern California with well-established and comprehensive electronic medical records (EMRs) and stable membership. Changes were reported by age, gender, race and ethnicity, estimated household income, weight status, and comorbidity subgroups. Weight status was included in this study because obesity affects 20% of US children and adolescents, and obesity is a risk factor for depression and anxiety.^15,16^ The relative strength of these factors with the incidence and prevalence of depression and anxiety diagnoses was also explored.

## Methods

### Study Population

This population-based cohort study included all members 5 to 22 years of age on January 1 of each calendar year from 2017 to 2021 within Kaiser Permanente Southern California (KPSC), an integrated health care system with more than 4.6 million members, representative of the Southern California population and regional diversity.^17,18^ We identified a total of 1 703 090 unique KPSC members who were aged 5 to 22 years during the study period; 1 339 549 (78.7%) had multiple years of KPSC membership, with approximately 1 million unique members per calendar year. Kaiser Permanente Southern California has a well-established comprehensive EMR system with social, demographic, and health care data. This study was approved by KPSC Institutional Review Boards, with individual participant consent waived because the research involved no more than minimal risk to participants and the research could not practicably be performed without the requested waiver. This study followed the Strengthening the Reporting of Observational Studies in Epidemiology (STROBE) reporting guideline for cohort studies.

### Clinical Diagnosis of Depression and Anxiety

A diagnosis of depression was identified based on clinical encounters with at least 1 code from the *International Statistical Classification of Diseases, Tenth Revision* (*ICD-10*), that corresponds with the American Psychiatric Association’s *Diagnostic and Statistical Manual of Mental Disorders* (Fifth Edition) criteria for the subclassification of MDD with single (F32.0-F32.3 and F32.9) or recurrent (F33.0-F33.3 and F33.9) episodes or persistent depressive disorder (dysthymic disorder) (F34.1).^10^ Anxiety was ascertained based on clinical encounters with at least 1 *ICD-10* code for anxiety states (F06.4, F40, F41, and F93.8), such as generalized anxiety disorder, social anxiety disorder, panic disorders, and other phobia-related disorders.^10^ Records from both inpatient and outpatient encounters were used. Prior studies have used these codes to identify depression and anxiety.^19,20^ All *ICD-10* codes are listed in eTable 1 in Supplement 1.

### Subgroups

The subgroups we assessed were categorized by age, demographic and economic measures, and health factors. Age was categorized into 5.0 to 10.9, 11.0 to 13.9, 14.0 to 17.9, and 18.0 to 22.9 years to approximate potentially different social environments corresponding to elementary school, middle school, high school, and college life in the US. Demographic and economic factors included gender (male or female), self-reported race and ethnicity (Hispanic, non-Hispanic American Indian or Alaska Native, non-Hispanic Asian, non-Hispanic Black, non-Hispanic Native Hawaiian or Other Pacific Islander, non-Hispanic White, non-Hispanic multiple races or ethnicities, non-Hispanic self-identified other, or unknown), and estimated household income based on census tract of residence ($0-$49 999, $50 000-$99 999, or≥$100 000). Race and ethnicity were assessed because prior studies showed these variables are associated with depression and anxiety.^1,21^ Health factors included weight status and comorbidity status. Weight status was defined using percentiles of body mass index (BMI; calculated as the weight in kilograms divided by the height in meters squared) for age and sex from the Centers for Disease Control and Prevention^22^ for 5 to 19 years of age and categorized as underweight (<5%), normal weight (5% to <85%), overweight (85% to <95%), and obesity (≥95%). Weight status for participants 20 years and older was defined by BMI and categorized as underweight (<18.5), normal weight (18.5 to <25.0), overweight (25.0 to <30.0), and obesity (≥30.0). To calculate BMI or BMI percentile, weight and height measurements from the corresponding calendar year were preferentially used; missingness was filled in by the last measurements from the year prior or the first measurements from the year after. History of comorbidity was defined as having any condition included in the Charlson Comorbidity Index,^23^ from the calendar year prior.

### Statistical Analysis

Data were analyzed from June 1, 2022, to November 29, 2023. To assess the incidence of depression by calendar year, members with a history of depression diagnosis prior to the calendar year were not included. To assess the incidence of anxiety without depression, members with a history of anxiety or depression diagnosis prior to the calendar year were excluded. Incidence for each calendar year and by subgroups was calculated as the number of new cases over the total number of participants at risk in the year and the corresponding subgroups. Prevalence was calculated as the number of cases over the total number of participants in the year and corresponding subgroups. Changes across calendar years were presented graphically and significance over time was tested by Poisson regression. Changes owing to the COVID-19 pandemic and difference in changes between the periods before (2017-2019) and during (2020-2021) the pandemic were assessed by including an indicator variable for COVID-19 (0 for 2017-2019 and 1 for 2020-2021) and testing for the main effect and interaction with time. Two-sided *P* < .05 was considered statistically significant.

Multivariable Poisson regression and deviance analyses were used to assess the relative strength of each subgroup factor with the incidence and prevalence of the outcomes for every calendar year. Changes in deviance between the full model and the model with removal of one variable at a time were used to assess the relative association, with a larger difference in the deviance indicating an association with the outcome. SAS Enterprise Guide, version 7.1 (SAS Institute Inc) was used for data analysis. All statistical tests were 2 sided.

## Results

The study included more than 1.1 million members aged 5 to 22 in each year from 2017 to 2021, with a total 1 703 090 unique participants. The mean (SD) age was approximately 14 (5) years; approximately 30% were aged 5.0 to 10.9 years, 16% were aged 11.0 to 13.9 years, 22% were aged 14.0 to 17.9 years, and 31% were aged 18.0 to 22.9 years during each study year (Table). Approximately 51% were male and 49% were female during each study year (Table). Across study years, approximately 50% were Hispanic, less than 1% were American Indian or Alaska Native, 8% were non-Hispanic Asian, 8% were non-Hispanic Black, 1% were Native Hawaiian or Other Pacific Islander, 23% were non-Hispanic White, and 2% were other; unknown race or ethnicity ranged from approximately 7% to 11%. The Table also presents the estimated household income, Medicaid or Children’s Health Insurance Program status, weight status, and history of comorbidity distribution for each year.

**Table.** Participant Characteristics

Characteristic	Calendar year^a^				
	2017 (n = 1 108 267)	2018 (n = 1 113 857)	2019 (n = 1 113 939)	2020 (n = 1 106 684)	2021 (n = 1 100 190)
Age, mean (SD), y	14.5 (5.2)	14.5 (5.2)	14.4 (5.2)	14.4 (5.2)	14.4 (5.2)
Age group, y					
5.0-10.9	334 913 (30.2)	338 845 (30.4)	339 127 (30.4)	336 116 (30.4)	334 137 (30.4)
11.0-13.9	176 105 (15.9)	179 361 (16.1)	183 245 (16.5)	184 820 (16.7)	181 556 (16.5)
14.0-17.9	249 887 (22.5)	247 968 (22.3)	246 977 (22.2)	246 660 (22.3)	247 219 (22.5)
18.0-22.9	347 362 (31.3)	347 683 (31.2)	344 590 (30.9)	339 088 (30.6)	337 278 (30.7)
Sex					
Female	542 548 (49.0)	545 737 (49.0)	545 887 (49.0)	542 108 (49.0)	539 102 (49.0)
Male	565 719 (51.0)	568 120 (51.0)	568 052 (51.0)	564 576 (51.0)	561 088 (51.0)
Race and ethnicity^b^					
American Indian or Alaska Native	1993 (0.2)	2027 (0.2)	1949 (0.2)	1932 (0.2)	1886 (0.2)
Asian	90 290 (8.1)	93 381 (8.4)	94 570 (8.5)	95 027 (8.6)	94 191 (8.6)
Black	88 241 (8.0)	86 465 (7.8)	84 704 (7.6)	81 822 (7.4)	79 548 (7.2)
Hispanic	559 965 (50.5)	553 060 (49.7)	546 453 (49.1)	534 379 (48.3)	521 176 (47.4)
Native Hawaiian or Other Pacific Islander	7422 (0.7)	7380 (0.7)	7226 (0.6)	6981 (0.6)	6711 (0.6)
White	259 335 (23.4)	260 688 (23.4)	257 835 (23.1)	251 937 (22.8)	244 957 (22.3)
Multiple	9157 (0.8)	9409 (0.8)	9463 (0.8)	9512 (0.9)	9341 (0.8)
Other^c^	17 931 (1.6)	20 034 (1.8)	22 041 (2.0)	23 605 (2.1)	24 266 (2.2)
Unknown	73 933 (6.7)	81 413 (7.3)	89 698 (8.1)	101 489 (9.2)	118 114 (10.7)
Estimated income					
$0-$49 999	319 382 (28.8)	262 236 (23.5)	231 336 (20.8)	180 182 (16.3)	146 604 (13.3)
$50 000-$99 999	614 087 (55.4)	620 465 (55.7)	622 911 (55.9)	623 189 (56.3)	615 808 (56.0)
≥$100 000	172 145 (15.5)	228 662 (20.5)	257 477 (23.1)	301 032 (27.2)	335 560 (30.5)
Missing	2653 (0.2)	2494 (0.2)	2215 (0.2)	2281 (0.2)	2218 (0.2)
Weight status					
Underweight	31 288 (2.8)	32 225 (2.9)	31 466 (2.8)	32 071 (2.9)	27 616 (2.5)
Normal	548 989 (49.5)	551 956 (49.6)	539 404 (48.4)	508 386 (45.9)	427 739 (38.9)
Overweight	179 984 (16.2)	179 554 (16.1)	174 667 (15.7)	170 962 (15.4)	149 538 (13.6)
Obese	200 448 (18.1)	203 839 (18.3)	202 463 (18.2)	209 082 (18.9)	199 502 (18.1)
Missing	147 558 (13.3)	146 283 (13.1)	165 939 (14.9)	186 183 (16.8)	295 795 (26.9)
Comorbidity the year prior					
No	1 009 408 (91.1)	1 011 536 (90.8)	1 013 416 (91.0)	1 006 254 (90.9)	1 023 132 (93.0)
Yes	98 859 (8.9)	102 321 (9.2)	100 523 (9.0)	100 430 (9.1)	77 058 (7.0)
Medicaid or CHIP coverage					
No	896 129 (80.9)	907 134 (81.4)	910 123 (81.7)	904 933 (81.8)	887 045 (80.6)
Yes	212 138 (19.1)	206 723 (18.6)	203 816 (18.3)	201 751 (18.2)	213 145 (19.4)

Abbreviation: CHIP, Children’s Health Insurance Program.

^a^ Unless otherwise indicated, data are expressed as No. (%) of participants. Percentages have been rounded and may not total 100.

^b^ Race and ethnicity are from participant self-report.

^c^ No further breakdown of this category is available.

**Figure 1.**
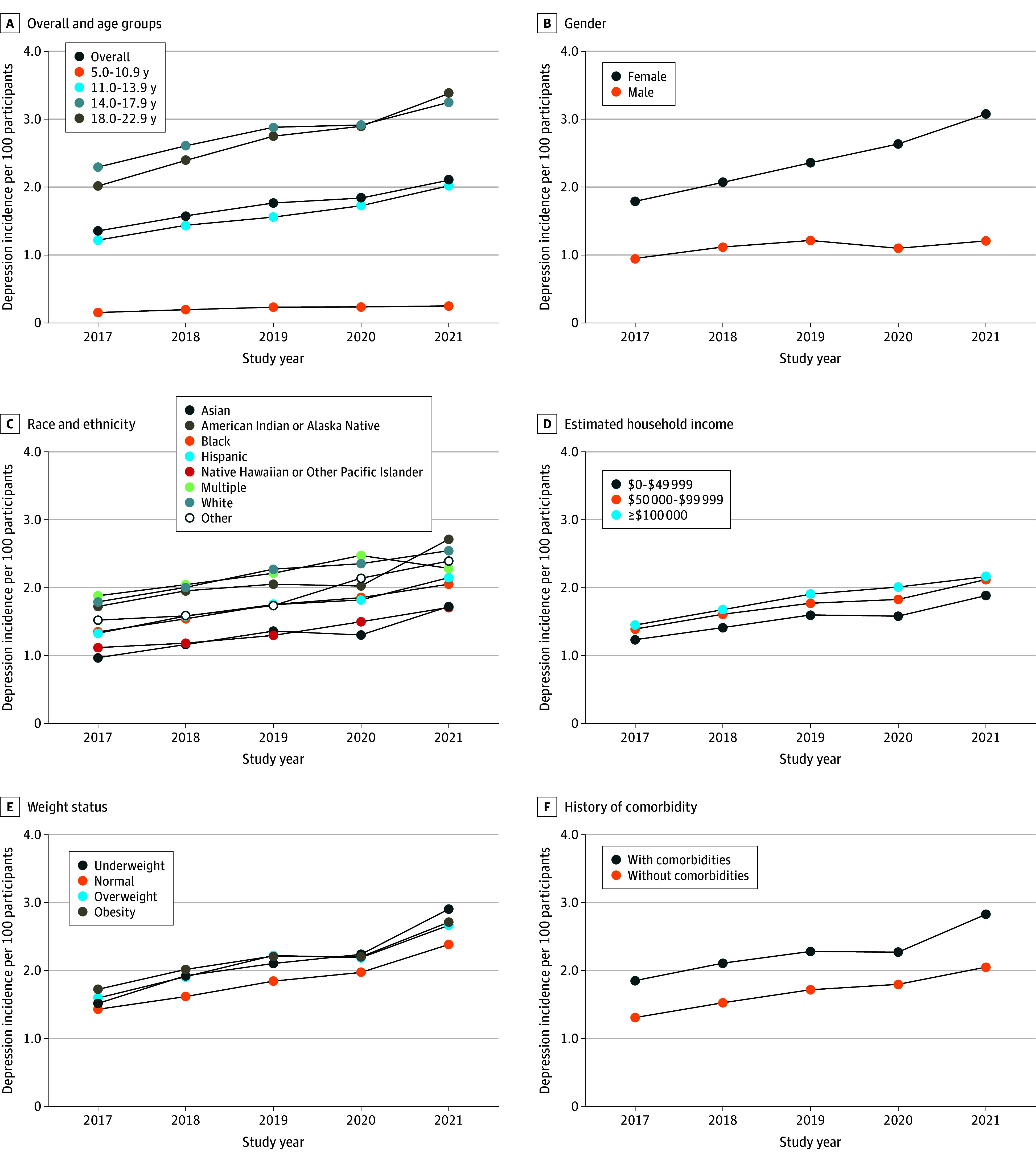
Incidence of Clinically Diagnosed Depression per 100 Participants From 2017 to 2021 Calculated as the number of new cases over the total number of participants at risk in the year and the corresponding subgroups.

**Figure 2.**
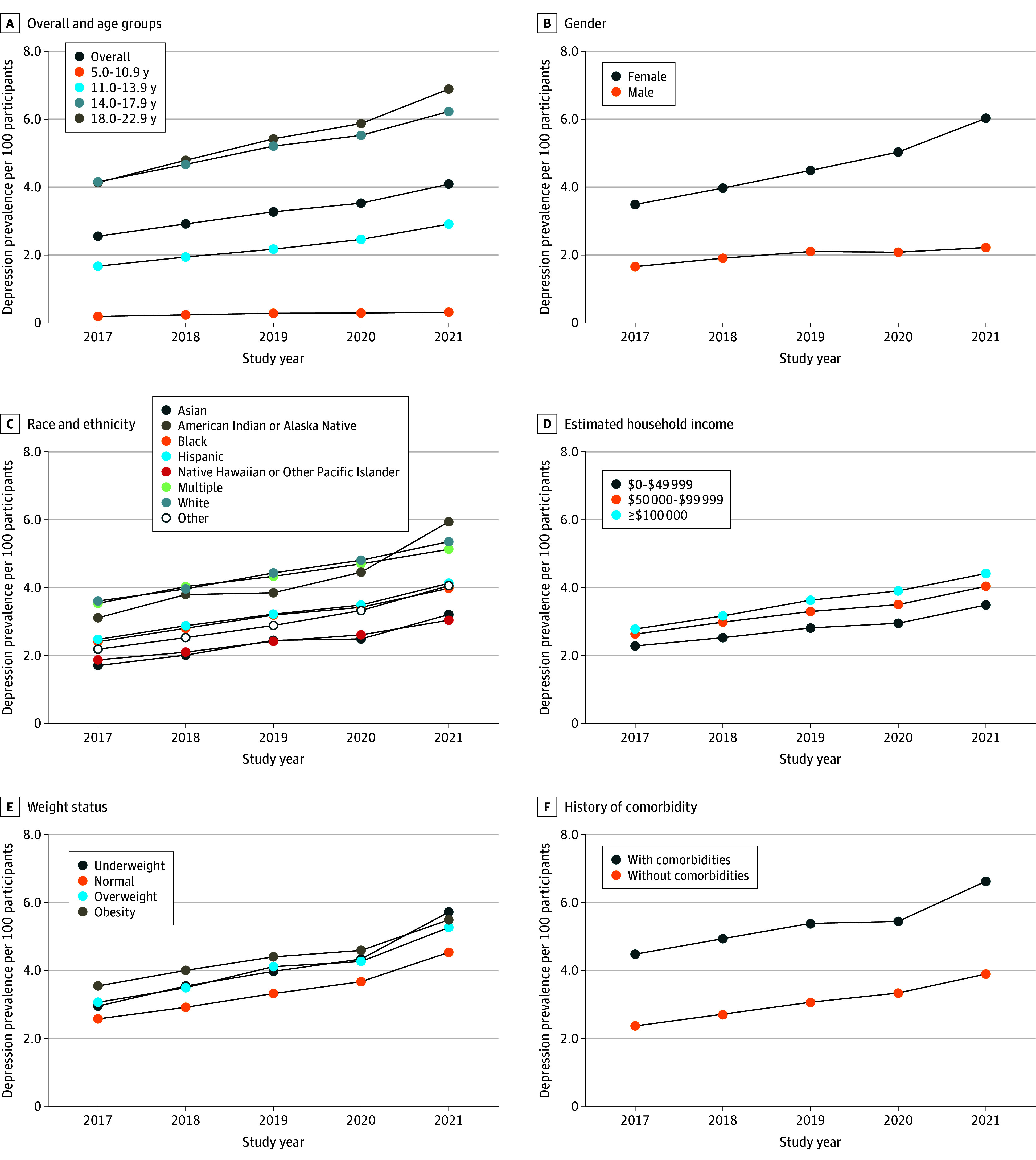
Prevalence of Clinically Diagnosed Depression per 100 Participants From 2017 to 2021 Calculated as the number of cases over the total number of participants in the year and corresponding subgroups.

The overall incidence of depression was 1.35% in 2017, 1.58% in 2018, 1.76% in 2019, 1.84% in 2020, and 2.10% in 2021, which significantly increased over the calendar years (55.6%; *P* < .001 for trend). This increase over time holds among different subgroups categorized by age, gender, race and ethnicity, estimated household income, weight, and comorbidity (Figure 1). In each of the calendar years, the incidence of depression was highest among those who were aged 14 to 17 and 18 to 22 years, female, of American Indian or Alaska Native (except for 2020) or non-Hispanic White race and ethnicity and who had higher estimated household income, obesity, and a history of comorbidities (Figure 1 and eTable 2 in Supplement 1 for detailed incidence by each year and subgroup). The mean incidence was higher during the COVID-19 pandemic than before the pandemic (1.97% [2020-2021] vs 1.56% [2017-2019]; *P* < .001) but the increase in rate from 2020-2021 (0.26%/y) was not significantly greater than the increase in rate from 2017-2019 (0.21%/y [*P* = .81]). Like the incidence, the prevalence of depression also significantly increased over the calendar years overall and among each of the subgroups (*P* < .001 for trend for all). For instance, the prevalence of depression increased from 4.13% in 2017 to 6.88% in 2021 (*P* < .001) among those aged 18.0 to 22.9 years (Figure 2 and eTable 3 in Supplement 1 for detailed rates). The overall prevalence of depression was 2.55% in 2017, 2.92% in 2018, 3.27% in 2019, 3.53% in 2020, and 4.08% in 2021 (60.0% increase; *P* < .001 for trend). The differences by subgroups were similar to the differences in the incidence by subgroups. Of note, the prevalence of depression for ages 5 to 17 years was 1.83% in 2017 and 2.85% in 2021. Meanwhile, for ages 14 to 17 years, the prevalence of depression was 4.15% in 2017 and 6.23% in 2021. The mean prevalence during the COVID-19 pandemic (3.81%) was higher than the prepandemic prevalence (2.91% [*P* < .001]), and the increase in rate during the pandemic (from 2020-2021) was greater than the increase in rate before the pandemic (2017-2019) (0.55%/y vs 0.36%/y [*P* = .004]).

**Figure 3.**
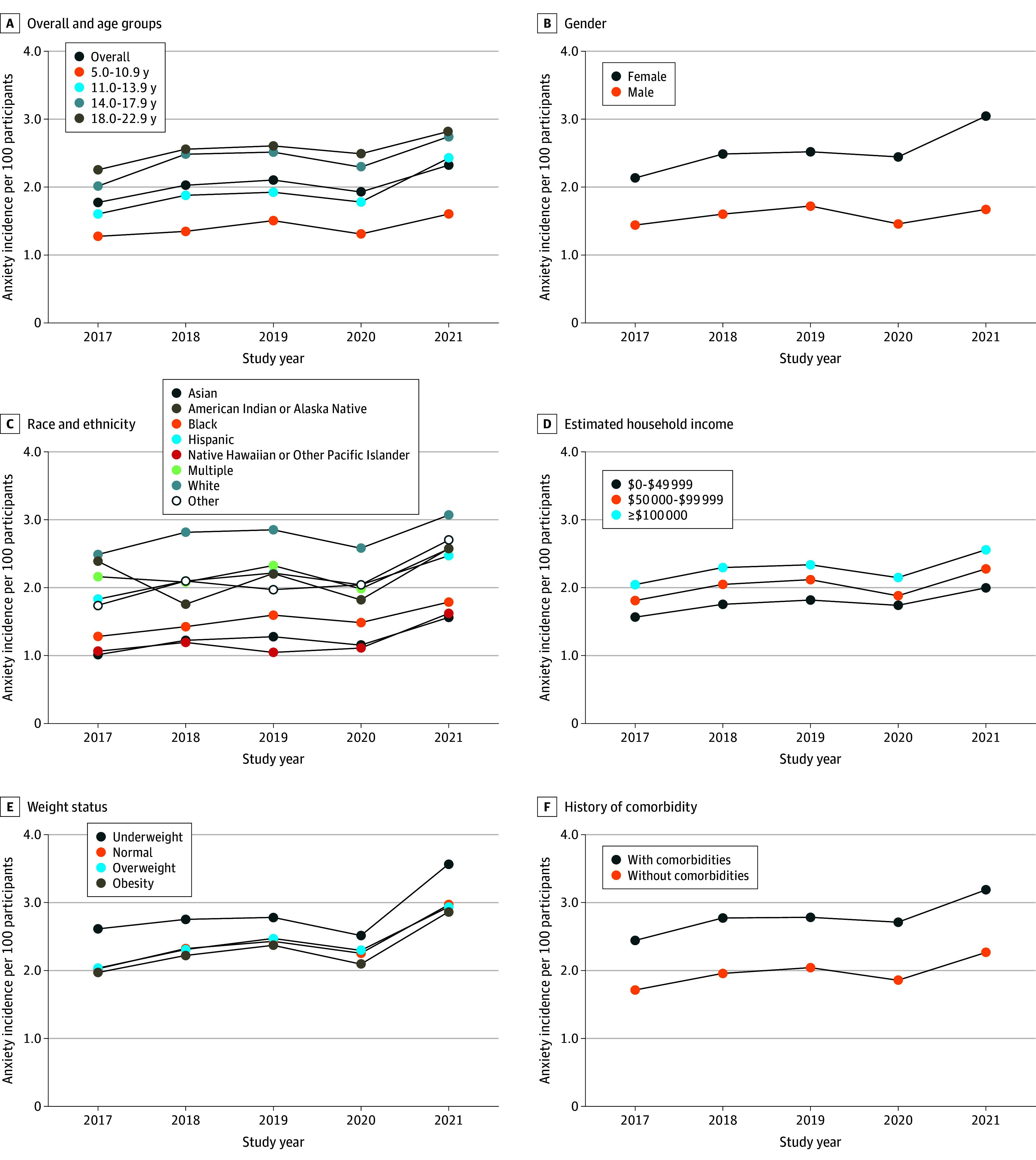
Incidence of Clinically Diagnosed Anxiety Without Depression per 100 Participants From 2017 to 2021 Calculated as the number of new cases over the total number of participants at risk in the year and the corresponding subgroups.

**Figure 4.**
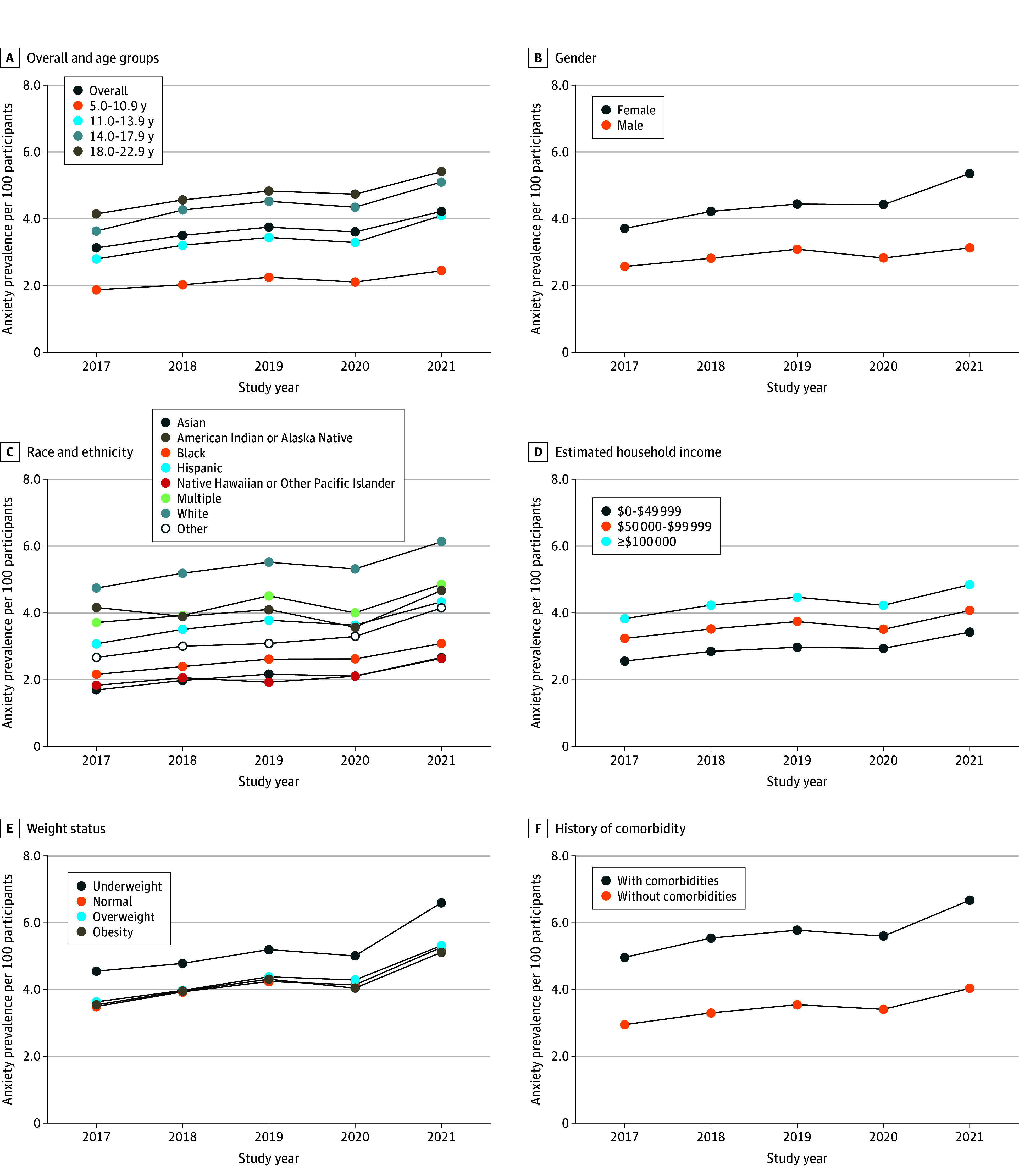
Prevalence of Clinically Diagnosed Anxiety Without Depression per 100 Participants From 2017 to 2021 Calculated as the number of cases over the total number of participants in the year and corresponding subgroups.

Among those with a diagnosis of anxiety, 36.5% individuals also had a depression diagnosis. We focused on only those who had anxiety without depression. The overall incidence of anxiety without depression was 1.77% in 2017, 2.03% in 2018, 2.10% in 2019, 1.93% in 2020, and 2.32% in 2021, representing a significant increase over the calendar years (31.1%), although there was a slight dip in 2020 (*P* < .001 for trend) (Figure 3). This pattern over time generally holds among the different subgroups (Figure 3 and eTable 4 in Supplement 1 for detailed rates). For all subgroups, the rates were highest in 2021 than any of the earlier years. The incidence of anxiety without depression increased with age and was highest in the subgroup aged 18 to 22 years; was higher in female than male participants (except in 2019), and was highest in those of non-Hispanic White race or with highest estimated household income, underweight, or a history of comorbidities. Similar to incidence, prevalence of anxiety without depression also significantly increased with calendar year overall to 3.13% in 2017, 3.51% in 2018, 3.75% in 2019, 3.61% in 2020, and 4.22% in 2021 (35.2% increase; *P* < .001 for trend) (Figure 4 and eTable 5 in Supplement 1 provide detailed rates). The differences in prevalence by subgroups were similar to the differences in incidence by subgroups (Figure 4). The means for both the incidence and prevalence were higher during the COVID-19 pandemic than before the pandemic (2.13% vs 1.97% [*P* < .001] for incidence and 3.92% vs 3.46% [*P* < .001] for prevalence), and the rates of change in the incidence and prevalence of anxiety without depression were greater during than before the pandemic (0.39%/y vs 0.17%/y [*P* < .001] for incidence and 0.61%/y vs 0.31%/y [*P* < .001] for prevalence).

When assessing the relative contribution of each of the subgroup factors to depression and anxiety, the following variables in the order of largest to the smallest were identified for the incidence of depression: age, weight status, gender, race and ethnicity, comorbidity, and estimated household income for 2017 to 2019 and age, gender, weight status, race and ethnicity, comorbidity, and estimated income for 2020 to 2021 (see eTable 6 in Supplement 1 for differences in deviance for each variable and outcome). This ranking was also observed for the prevalence of depression (eTable 6 in Supplement 1). The corresponding ranking of the variables for the incidence of anxiety without depression were as follows: weight status, age, race and ethnicity, gender, comorbidity, and estimated income for 2017 to 2019 and weight status, age, gender, race and ethnicity, comorbidity, and estimated income for 2020 to 2021 (eTable 6 in the Supplement). The ranking for the prevalence of anxiety were as follows: weight status, age, race and ethnicity, gender, comorbidity, and estimated income for each of the calendar year from 2017 to 2021 (eTable 6 in Supplement 1). Of note, all these factors were independently associated with each of the outcomes for each year, when testing for association in Poisson regression for all variables in each year, except for income with incidence of depression (eTable 7 in Supplement 1).

## Discussion

Our cohort study population, composed of more than 1.7 million individuals aged 5 to 22 years, is representative of the Southern California general population^17^ and showed that incidence and prevalence of clinically diagnosed depression and anxiety without depression significantly increased from 2017 to 2021. The increase in rates was generally higher during the COVID-19 pandemic (2020-2021) than before the pandemic (2017-2019). The significant increases were observed across all age, gender, race and ethnicity, estimated household income, weight status, and comorbidity subgroups. Rates of depression were higher for those aged 14 to 17 and 18 to 22 years; female participants; those of American Indian or Alaska Native, non-Hispanic White, or multiple races and ethnicities; and those with higher household income, obesity, or comorbidities. Similar subgroup differences were observed for anxiety without depression except that the group with underweight had higher incidence and prevalence of anxiety without depression. Exploratory data analysis on the relative importance among the 6 demographic, economic, and health factors identified age, weight status, gender, and race and ethnicity as the most important indicators contributing to differences in rates of depression and anxiety without depression. Age appeared to be the most important factor for depression; however, weight status was the most important factor for anxiety without depression.

The overall incidence of depression diagnosed clinically increased by 55.6% over the 5-year period and prevalence of depression clinically diagnosed increased by 60.0%. To our knowledge, no large population-based studies have assessed the incidence of depression in children, adolescents, and young adults. The prevalence of depression in our study for the subgroup aged 5 to 17 years was 1.83% in 2017 and 2.85% in 2021, which is lower than the 3.1% in 2016 and 4.0% in 2020 from the NSCH survey report.^4^ Our prevalence of depression for the subgroup aged 14 to 17 years was 6.23% in 2021, lower than the 20% reported from the 2021 NSDUH survey report.^5,6^ The lower rate in clinical diagnosis is likely due to some youths having episodes of depression who were not diagnosed or not seen by health care professionals, as reported from the NSDUH study that more than half of the youths with MDD did not have any mental health treatment.^5,6^ However, our data were in line with the NSCH 2016-2019 estimate of 2.1% current depression in youths aged 3 to 17 years in California.^1^ Recently, Visser et al^24^ compared rates of parent-reported attention-deficit/hyperactivity disorder to the rate of attention-deficit/hyperactivity disorder diagnosis ascertained by EMR data in Southern California and found the rates to be comparable (4.7% vs 4.9%), suggesting convergent validity between parent report and EMR diagnosis.

For anxiety without depression, we observed an increase of 31.1% for incidence and 35.2% for prevalence. Data from NSCH reported a 29% increase in the prevalence of overall anxiety.^4^ However, anxiety without depression was not separately reported.

Among subgroups, higher incidence and prevalence of depression and anxiety without depression were observed among adolescents and young adults; female participants; those of non-Hispanic American Indian or Alaska Native, non-Hispanic White, or multiple races or ethnicities; and those with underweight and obesity, higher household income, and comorbidities. Our findings are consistent with previous survey data that showed higher rates of depression or anxiety among girls, older children and adolescents, those of White race or ethnicity,^1,2,4,7,25,26,27,28,29^ and those of multiple races or ethnicities.^5^ Obesity has been consistently shown as a risk factor for depression. A previous meta-analysis^15^ showed that children and adolescents younger than 21 years with obesity had a 34% higher risk of developing depression than their peers with healthy weight. However, unlike prior survey data, we found higher rates of depression or anxiety among youths from families with higher incomes.^1,7,12,25^ The subgroup with underweight had the highest incidence and prevalence of anxiety without a depression diagnosis. Our data showed that among the factors we assessed, weight status was the most important factor (higher than gender, age, and race and ethnicity) contributing to risk of anxiety without depression. Although underweight was less studied in the literature, previous studies indicated that weight-related teasing for adolescents with underweight (and overweight) may increase the risk of social isolation and mental health problems for youths and adolescents.^30,31^ Other studies have shown that individuals with eating disorders often have comorbid mood or anxiety disorders.^32,33^ The group with underweight may benefit from early interventions to reduce anxiety to prevent future development of depression.

The unexpected onset of the COVID-19 pandemic in 2020 disrupted many health care systems and their services. It also contributed to an increase in mental health issues for adolescents.^34^ Our data showed that the rates of newly diagnosed clinical depression did not decrease because of problems with access to health care in 2020, indicating that patients with depression or anxiety were still receiving care. Instead, the COVID-19 pandemic contributed to the already increasing trends. The rate continued to increase in 2021, at which point it was the highest among the years we assessed. This increase in newly diagnosed depression was observed for all subgroups. For anxiety without depression, there was a slight decrease in 2020; however, 2021 has the highest incidence among the years we assessed. A population-based study in Canada that included children aged 3 to 17 years from January 1, 2017, to February 28, 2021,^35^ showed that mental health visits declined during the first quarter of the pandemic, followed by a rapid increase in outpatient visits and virtual care. Depression and anxiety were studied along with other conditions and collapsed into a broader category of mood disorders; rates of each condition were not provided.^35^

### Strengths and Limitations

Our study has several important strengths. To our knowledge, this is the first large population-based, multiethnic study that assessed the changes in the incidence and prevalence of clinically diagnosed depression and anxiety without depression among children, adolescents, and young adults in the US for a period including both before and during the COVID-19 pandemic. Rates of depression and anxiety were separately assessed during childhood, adolescence, and young adulthood, which corresponds to school environments, and our results revealed important differences among these different developmental periods. We included several demographic, economic, and health factors to assess potential disparities and explored the importance of the factors on the risk of depression and anxiety. Depression is a common comorbidity associated with anxiety; instead of assessing anxiety overall, we focused on anxiety without depression, a phenotype that may not have drawn a lot of attention in clinical care and public health attention. All data came from a single integrated health care system with uniform care standards and guidelines, including those implemented during the COVID-19 pandemic; thus, minimizing potential screening and classification biases.

We recognize that our study has some limitations. Although our study population is representative of the general population in Southern California, it may not be generalizable to the US population overall.^17^ However, the major limitation is that this study used clinical diagnosis of depression and anxiety. There may be barriers to clinical diagnosis, such as differential expression of symptoms in children; the severity of symptoms may limit appointment attendance; or limited health care access could prevent children, adolescents, and young adults from obtaining formal diagnoses. In addition, structural and cultural barriers to mental health care such as perceived need for care, mental health illness stigma, lack of diversity in mental health care professionals, cultural competence among clinicians, and distrust in the health care system were not addressed in this study.^36,37^ Thus, the data used herein may underrepresent the true prevalence and incidence of depression and anxiety, as shown in surveys. Considering the rising rates of both conditions reported herein, potential underreporting of those with mental health conditions only further indicates the need for strengthened efforts to identify and support young people with depression and anxiety.

## Conclusions

In this large, multiethnic population-based cohort study, the incidence and prevalence of clinically diagnosed depression and anxiety without depression significantly increased over time from 2017 to 2021 and from before to during the COVID-19 period for children, adolescents, and young adults. The significant increases were observed across all age, gender, race and ethnicity, estimated household income, weight status, and comorbidity subgroups. Rates were higher for adolescents and young adults and in some subgroups, highlighting a need for increased mental health services for our young population.

## Notes

Supplement 1. **eTable 1.** *ICD-10* Codes Used for Depression and Anxiety **eTable 2.** Incidence of Depression (per 100 Person-Years) by Subgroups **eTable 3.** Prevalence of Depression (per 100 Person-Years) by Subgroups **eTable 4.** Incidence of Anxiety Without Depression (per 100 Person-Years) by Subgroups **eTable 5.** Prevalence of Anxiety Without Depression (per 100 Person-Years) by Subgroups **eTable 6.** Difference in Deviance of the Full Model and 1 Covariate Removed, by Calendar Year **eTable 7.** Associations Between Each Covariate and Outcomes Using Poisson Regression

Supplement 2.
Data Sharing Statement
