# Supplementary material for: Depression and Anxiety Among US Children and Young Adults
Source: JAMA Netw Open. 2024 Oct 1;7(10):e2436906. doi: 10.1001/jamanetworkopen.2024.36906 (PMC11445688; doi:10.1001/jamanetworkopen.2024.36906)
Supplement: Supplement 1. — eTable 1. ICD-10 Codes Used for Depression and Anxiety eTable 2. Incidence of Depression (per 100 Person-Years) by Subgroups eTable 3. Prevalence of Depression (per 100 Person-Years) by Subgroups eTable 4. Incidence of Anxiety Without Depression (per 100 Person-Years) by Subgroups eTable 5. Prevalence of Anxiety Without Depression (per 100 Person-Years) by Subgroups eTable 6. Difference in Deviance of the Full Model and 1 Covariate Removed, by Calendar Year eTable 7. Associations Between Each Covariate and Outcomes Using Poisson Regression [file jamanetwopen-e2436906-s001.pdf]

Supplementary Online Content

Xiang AH, Martinez MP, Chow T, et al. Depression and anxiety among US children and young adults. *JAMA Netw Open*. 2024;7(10):e2436906.
doi:10.1001/jamanetworkopen.2024.36906

eTable 1. *ICD-10* Codes Used for Depression and Anxiety
eTable 2. Incidence of Depression (per 100 Person-Years) by Subgroups
eTable 3. Prevalence of Depression (per 100 Person-Years) by Subgroups
eTable 4. Incidence of Anxiety Without Depression (per 100 Person-Years) by Subgroups
eTable 5. Prevalence of Anxiety Without Depression (per 100 Person-Years) by Subgroups
eTable 6. Difference in Deviance of the Full Model and 1 Covariate Removed, by Calendar Year
eTable 7. Associations Between Each Covariate and Outcome Using the Poisson Regression

This supplementary material has been provided by the authors to give readers additional information about their work.

© 2024 Xiang AH et al. *JAMA Network Open*.

eTable 1. *ICD-10* Codes Used for Depression and Anxiety

**Depression**	
**ICD 10 codes**	**Definition**
F32.9	Major depressive disorder, single episode, unspecified
F32.0	Major depressive disorder, single episode, mild
F32.1	Major depressive disorder, single episode, moderate
F32.2	Major depressive disorder, single episode, severe without psychotic features
F32.3	Major depressive disorder, single episode, severe with psychotic features
F33.9	Major depressive disorder, recurrent, unspecified
F33.0	Major depressive disorder, recurrent, mild
F33.1	Major depressive disorder, recurrent, moderate
F33.2	Major depressive disorder, recurrent severe without psychotic features
F33.3	Major depressive disorder, recurrent, severe with psychotic symptoms
F34.1	Dysthymic disorder
**Anxiety**	
**ICD 10 codes**	**Definition**
F06.4	Anxiety disorder due to known physiological condition
F40.00	Agoraphobia, unspecified
F40.01	Agoraphobia with panic disorder
F40.02	Agoraphobia without panic disorder
F40.10	Social phobia, unspecified
F40.11	Social phobia, generalized
F40.210	Arachnophobia
F40.218	Other animal type phobia
F40.220	Fear of thunderstorms
F40.228	Other natural environment type phobia
F40.230	Fear of blood
F40.231	Fear of injections and transfusions
F40.232	Fear of other medical care
F40.233	Fear of injury
F40.240	Claustrophobia
F40.241	Acrophobia
F40.242	Fear of bridges
F40.243	Fear of flying
F40.248	Other situational type phobia
F40.290	Androphobia
F40.291	Gynephobia
F40.298	Other specified phobia
F40.8	Other phobic anxiety disorders
F40.9	Phobic anxiety disorder, unspecified
F41.0	Panic disorder [episodic paroxysmal anxiety]
F41.1	Generalized anxiety disorder

© 2024 Xiang AH et al. *JAMA Network Open*.

F41.3	Other mixed anxiety disorders
F41.8	Other specified anxiety disorders
F41.9	Anxiety disorder, unspecified
F93.8	Other childhood emotional disorders

© 2024 Xiang AH et al. *JAMA Network Open*.

eTable 2. Incidence of Depression (per 100 Person-Years) by Subgroups

	By Calendar Year				
**Calendar Year**<br>**N (%)**	**2017**<br>**N=1,050,016**	**2018**<br>**N=1,053,974**	**2019**<br>**N=1,050,852**	**2020**<br>**N=1,039,305**	**2021**<br>**N=1,028,470**
**Age (years)** (%) *					
Overall	1.35	1.58	1.76	1.84	2.10
5-10.9	0.15	0.19	0.23	0.23	0.25
11-13.9	1.22	1.44	1.56	1.73	2.02
14-17.9	2.29	2.61	2.88	2.91	3.25
18-22.9	2.02	2.39	2.75	2.89	3.38
**Sex (%)**					
Female	1.79	2.07	2.36	2.63	3.07
Male	0.95	1.12	1.21	1.10	1.21
**Race and Ethnicity (%)**					
Hispanic	1.33	1.58	1.75	1.82	2.15
White	1.79	2.00	2.27	2.35	2.54
Black	1.35	1.54	1.75	1.85	2.05
Asian	0.97	1.16	1.36	1.30	1.72
Pacific Islander	1.12	1.18	1.30	1.50	1.71
Native American or Alaskan	1.72	1.95	2.05	2.02	2.71
Multiple	1.88	2.04	2.21	2.47	2.28
Other	1.52	1.59	1.73	2.14	2.39
Unknown	0.48	0.72	0.90	1.10	1.33
**Estimated Income (%)**					
$0-50,000	1.23	1.41	1.60	1.58	1.88
$50,000-100,000	1.39	1.61	1.77	1.83	2.12
>=$100,000	1.45	1.68	1.90	2.01	2.16

© 2024 Xiang AH et al. *JAMA Network Open*.

Missing	1.15	1.61	1.50	2.17	2.38
**Weight Status (%)**					
Underweight	1.51	1.92	2.10	2.24	2.90
Normal	1.43	1.62	1.84	1.97	2.38
Overweight	1.59	1.91	2.22	2.19	2.66
Obese	1.72	2.02	2.21	2.20	2.71
Missing	0.29	0.38	0.47	0.71	0.96
**Comorbidity the Year Prior (%)**					
No	1.31	1.52	1.72	1.80	2.05
Yes	1.85	2.11	2.28	2.27	2.83

© 2024 Xiang AH et al. *JAMA Network Open*.

eTable 3. Prevalence of Depression (per 100 Person-Years) by Subgroups

	By Calendar Year				
**Calendar Year N (%)**	**2017 N=1,108,267**	**2018 N=1,113,857**	**2019 N=1,113,939**	**2020 N=1,106,684**	**2021 N=1,100,190**
**Age (years)** (%) *					
Overall	2.55	2.92	3.27	3.53	4.08
5-10.9	0.19	0.23	0.28	0.29	0.31
11-13.9	1.67	1.94	2.17	2.46	2.91
14-17.9	4.15	4.67	5.20	5.52	6.23
18-22.9	4.13	4.79	5.42	5.87	6.88
**Sex (%)**					
Female	3.49	3.97	4.49	5.03	6.02
Male	1.66	1.9	2.1	2.08	2.22
**Race and Ethnicity (%)**					
Hispanic	2.48	2.88	3.22	3.49	4.13
White	3.61	3.97	4.43	4.81	5.35
Black	2.41	2.80	3.19	3.42	3.98
Asian	1.71	2.01	2.45	2.49	3.21
Pacific Islander	1.87	2.10	2.42	2.61	3.04
Native American or Alaskan	3.11	3.80	3.85	4.45	5.94
Multiple	3.54	4.03	4.33	4.70	5.13
Other	2.19	2.53	2.89	3.33	4.06
Unknown	0.69	1.01	1.27	1.57	1.99
**Estimated Income (%)**					
$0-50,000	2.28	2.52	2.81	2.95	3.49
$50,000-100,000	2.63	2.99	3.30	3.50	4.04
>=$100,000	2.78	3.17	3.63	3.91	4.42
Missing	2.49	3.29	3.57	5.35	6.18

© 2024 Xiang AH et al. *JAMA Network Open*.

**Weight Status (%)**					
Underweight	2.95	3.54	3.97	4.33	5.72
Normal	2.57	2.91	3.32	3.67	4.53
Overweight	3.06	3.49	4.11	4.26	5.26
Obese	3.54	4.00	4.40	4.59	5.49
Missing	0.43	0.56	0.73	1.13	1.74
**Comorbidity the Year Prior (%)**					
No	2.37	2.71	3.06	3.34	3.89
Yes	4.48	4.94	5.39	5.44	6.62

© 2024 Xiang AH et al. *JAMA Network Open*.

eTable 4. Incidence of Anxiety Without Depression (per 100 Person-Years) by Subgroups

	By Calendar Year				
**Calendar Year N (%)**	**2017 N=997,541**	**2018 N=995,015**	**2019 N=984,279**	**2020 N=965,864**	**2021 N=950,427**
**Age (years)***					
Overall (%)	1.77	2.03	2.10	1.93	2.32
Age group (%)					
5-10.9	1.27	1.35	1.51	1.31	1.60
11-13.9	1.60	1.88	1.92	1.78	2.43
14-17.9	2.01	2.48	2.52	2.29	2.74
18-22.9	2.25	2.56	2.61	2.49	2.82
**Sex (%)**					
Female	2.13	2.49	2.52	2.44	3.04
Male	1.44	1.60	1.72	1.46	1.67
**Race and Ethnicity (%)**					
Hispanic	1.83	2.10	2.22	2.04	2.47
White	2.49	2.82	2.85	2.58	3.07
Black	1.28	1.42	1.60	1.49	1.79
Asian	1.01	1.23	1.28	1.16	1.56
Pacific Islander	1.06	1.19	1.05	1.11	1.62
Native American or Alaskan	2.39	1.76	2.21	1.82	2.57
Multiple	2.16	2.08	2.33	1.98	2.57
Other	1.74	2.10	1.97	2.04	2.70
Unknown	0.62	0.91	0.97	1.04	1.28
**Estimated Income (%)**					
$0-50,000	1.56	1.75	1.82	1.74	2.00
$50,000-100,000	1.81	2.05	2.12	1.88	2.28
>=$100,000	2.04	2.30	2.34	2.15	2.56

© 2024 Xiang AH et al. *JAMA Network Open*.

Missing	1.32	1.51	1.50	1.75	2.33
**Weight Status (%)**					
Underweight	2.61	2.75	2.78	2.52	3.57
Normal	2.03	2.32	2.43	2.26	2.97
Overweight	2.03	2.31	2.47	2.30	2.94
Obese	1.97	2.22	2.37	2.09	2.87
Missing	0.18	0.26	0.35	0.53	0.72
**Comorbidity the Year Prior (%)**					
No	1.71	1.96	2.04	1.86	2.26
Yes	2.44	2.77	2.78	2.71	3.19

© 2024 Xiang AH et al. *JAMA Network Open*.

eTable 5. Prevalence of Anxiety Without Depression (per 100 Person-Years) by Subgroups

	By Calendar Year				
**Calendar Year**<br>**N (%)**	**2017**<br>**N=1,108,267**	**2018**<br>**N=1,113,857**	**2019**<br>**N=1,113,939**	**2020**<br>**N=1,106,684**	**2021**<br>**N=1,100,190**
**Age (years) (%) ***					
Overall	3.13	3.51	3.75	3.61	4.22
5-10.9	1.87	2.02	2.25	2.10	2.44
11-13.9	2.80	3.21	3.44	3.29	4.09
14-17.9	3.63	4.26	4.52	4.35	5.10
18-22.9	4.15	4.56	4.83	4.74	5.41
**Sex (%)**					
Female	3.71	4.22	4.44	4.42	5.35
Male	2.57	2.82	3.09	2.83	3.13
**Race and Ethnicity (%)**					
Hispanic	3.08	3.51	3.78	3.64	4.33
White	4.74	5.19	5.52	5.32	6.12
Black	2.16	2.39	2.62	2.63	3.08
Asian	1.69	1.98	2.17	2.11	2.66
Pacific Islander	1.83	2.06	1.92	2.11	2.64
Native American or Alaskan	4.16	3.90	4.10	3.57	4.67
Multiple	3.71	3.92	4.51	4.01	4.85
Other	2.67	3.00	3.09	3.30	4.15
Unknown	0.92	1.25	1.41	1.55	1.86
**Estimated Income (%)**					
$0-50,000	2.56	2.85	2.97	2.94	3.42
$50,000-100,000	3.24	3.52	3.74	3.51	4.07
>=$100,000	3.83	4.23	4.47	4.23	4.84
Missing	2.45	2.33	3.30	3.16	4.55

© 2024 Xiang AH et al. *JAMA Network Open*.

**Weight Status (%)**					
Underweight	4.55	4.78	5.19	5.01	6.60
Normal	3.49	3.93	4.24	4.14	5.26
Overweight	3.63	3.97	4.38	4.29	5.31
Obese	3.54	3.95	4.31	4.04	5.11
Missing	0.31	0.42	0.54	0.82	1.34
**Comorbidity the Year Prior (%)**					
No	2.95	3.3	3.55	3.41	4.04
Yes	4.97	5.54	5.78	5.60	6.68

© 2024 Xiang AH et al. *JAMA Network Open*.

eTable 6. Difference in Deviance of the Full Model and 1 Covariate Removed, by Calendar Year

	Difference in Deviance, by Calendar Year				
Model	**2017**	**2018**	**2019**	**2020**	**2021**
Incidence of Depression					
**Covariate Removed**					
Comorbidity	132.4	129.0	90.6	80.5	85.1
Obesity	1700.2	1955.3	2335.5	1847.3	2939.3
Estimated Income	11.2	24.1	16.0	53.6	19.8
Race/Ethnicity	499.9	407.3	472.3	529.6	358.5
Sex	1195.0	1339.3	1725.3	3128.7	3996.4
Age	8533.7	9669.9	10807.3	11189.3	13160.9
Prevalence of Depression					
**Covariate Removed**					
Comorbidity	1026.1	992.2	863.0	718.2	568.8
Obesity	3655.9	3997.9	4694.7	4172.2	5950.8
Estimated Income	37.8	84.4	106.0	154.8	124.2
Race/Ethnicity	1461.8	1284.8	1301.1	1513.5	1352.7
Sex	3001.5	3385.1	4017.9	5892.6	8442.4
Age	18925.3	21413.8	23842.5	25655.6	29988.2
Incidence of Anxiety without Depression					
**Covariate Removed**					
Comorbidity	151.4	171.8	103.2	187.7	72.7
Obesity	3058.3	3272.0	3431.5	2518.9	4694.4
Estimated Income	45.7	53.2	59.9	48.0	65.4
Race/Ethnicity	1058.2	1105.9	1030.3	895.0	867.6
Sex	533.9	797.1	602.4	1070.4	1683.9
Age	1505.1	2090.5	1742.5	1980.4	2040.7

© 2024 Xiang AH et al. *JAMA Network Open*.

Prevalence of Anxiety without Depression

Covariate Removed

Comorbidity	747.9	831.2	675.2	702.9	478.7
Obesity	5389.9	5566.7	6440.2	5337.1	8246.8
Estimated Income	230.8	263.9	324.8	222.7	234.3
Race/Ethnicity	2741.6	2767.3	2896.7	2659.0	2834.6
Sex	801.8	1165.6	951.7	1481.8	2446.7
Age	4160.2	4872.9	4756.5	5076.9	5658.2

© 2024 Xiang AH et al. *JAMA Network Open*.

eTable 7. Associations Between Each Covariate and Outcome Using Poisson Regression

		2017			2018			2019			2020			2021		
**Outcome**	**Covariate**	**DF**	**Chi-Square**	**P-value**	**DF**	**Chi-Square**	**P-value**	**DF**	**Chi-Square**	**P-value**	**DF**	**Chi-Square**	**P-value**	**DF**	**Chi-Square**	**P-value**
**Depression Incidence**	Age Groups	3	8533.69	<.001	3	9669.87	<.001	3	10807.3	<.001	3	11189.3	<.001	3	13160.9	<.001
	Gender	1	1195.01	<.001	1	1339.3	<.001	1	1725.26	<.001	1	3128.66	<.001	1	3996.39	<.001
	Race and Ethnicity	8	499.93	<.001	8	407.27	<.001	8	472.34	<.001	8	529.63	<.001	8	358.51	<.001
	Income	3	11.22	0.0106	3	24.06	<.001	3	16.02	0.0011	3	53.58	<.001	3	19.77	<.001
	Weight Status	4	1700.17	<.001	4	1955.27	<.001	4	2335.48	<.001	4	1847.34	<.001	4	2939.26	<.001
	History of Comorbidity	1	132.35	<.001	1	129.01	<.001	1	90.58	<.001	1	80.5	<.001	1	85.14	<.001
**Anxiety Incidence**	Age Groups	3	1505.07	<.001	3	2090.47	<.001	3	1742.47	<.001	3	1980.37	<.001	3	2040.73	<.001
	Gender	1	533.94	<.001	1	797.09	<.001	1	602.42	<.001	1	1070.42	<.001	1	1683.91	<.001
	Race and Ethnicity	8	1058.24	<.001	8	1105.87	<.001	8	1030.32	<.001	8	895.02	<.001	8	867.62	<.001
	Income	3	45.67	<.001	3	53.16	<.001	3	59.92	<.001	3	48.01	<.001	3	65.4	<.001
	Weight Status	4	3058.29	<.001	4	3271.98	<.001	4	3431.53	<.001	4	2518.95	<.001	4	4694.42	<.001
	History of Comorbidity	1	151.39	<.001	1	171.81	<.001	1	103.18	<.001	1	187.69	<.001	1	72.74	<.001
**Depression Prevalence**	Age Groups	3	18925.3	<.001	3	21413.8	<.001	3	23842.5	<.001	3	25655.6	<.001	3	29988.2	<.001
	Gender	1	3001.48	<.001	1	3385.11	<.001	1	4017.95	<.001	1	5892.59	<.001	1	8442.39	<.001
	Race and Ethnicity	8	1461.81	<.001	8	1284.81	<.001	8	1301.12	<.001	8	1513.53	<.001	8	1352.73	<.001
	Income	3	37.76	<.001	3	84.35	<.001	3	105.97	<.001	3	154.77	<.001	3	124.16	<.001

© 2024 Xiang AH et al. *JAMA Network Open*.

	Weight Status	4	3655.87	<.001	4	3997.85	<.001	4	4694.69	<.001	4	4172.21	<.001	4	5950.75	<.001
	History of Comorbidity	1	1026.09	<.001	1	992.2	<.001	1	863.03	<.001	1	718.17	<.001	1	568.75	<.001
**Anxiety Prevalence**	Age Groups	3	4160.2	<.001	3	4872.9	<.001	3	4756.54	<.001	3	5076.91	<.001	3	5658.17	<.001
	Gender	1	801.82	<.001	1	1165.59	<.001	1	951.65	<.001	1	1481.85	<.001	1	2446.67	<.001
	Race and Ethnicity	8	2741.62	<.001	8	2767.29	<.001	8	2896.73	<.001	8	2658.96	<.001	8	2834.56	<.001
	Income	3	230.83	<.001	3	263.86	<.001	3	324.82	<.001	3	222.69	<.001	3	234.35	<.001
	Weight Status	4	5389.93	<.001	4	5566.71	<.001	4	6440.17	<.001	4	5337.15	<.001	4	8246.79	<.001
	History of Comorbidity	1	747.94	<.001	1	831.21	<.001	1	675.18	<.001	1	702.94	<.001	1	478.66	<.001

*Income= Estimated household income ; Chi-square was based on the likelihood ratio test

© 2024 Xiang AH et al. *JAMA Network Open*.
