# Supplementary material for: Depression and Anxiety Among US Children and Young Adults
Source: JAMA Netw Open. 2024 Oct 1;7(10):e2436906. doi: 10.1001/jamanetworkopen.2024.36906 (PMC11445688; doi:10.1001/jamanetworkopen.2024.36906)
Supplement: Supplement 2. — Data Sharing Statement [file jamanetwopen-e2436906-s002.pdf]

Data Sharing Statement

Xiang. Depression and Anxiety Among US Children and Young Adults. *JAMA Netw Open*. Published October 01, 2024. doi:10.1001/jamanetworkopen.2024.36906

Data
Data available: No

Additional Information
Explanation for why data not available: The individual level data is not available to others as the data is drawn from electronic medical records.
